# Contextualizing Obesity and Diabetes Policy: Exploring a Nested Statistical and Constructivist Approach at the Cross-National and Subnational Government Level in the United States and Brazil

**DOI:** 10.15171/ijhpm.2017.13

**Published:** 2017-02-27

**Authors:** Eduardo J. Gómez

**Affiliations:** Department of International Development, School of Global Affairs, King’s College London, London, UK.

**Keywords:** Obesity, Diabetes, Policy-making, Subnational Government

## Abstract

**Background:** This article conducts a comparative national and subnational government analysis of the political, economic, and ideational constructivist contextual factors facilitating the adoption of obesity and diabetes policy.

**Methods:** We adopt a nested analytical approach to policy analysis, which combines cross-national statistical analysis with subnational case study comparisons to examine theoretical prepositions and discover alternative contextual factors; this was combined with an ideational constructivist approach to policy-making.

**Results:** Contrary to the existing literature, we found that with the exception of cross-national statistical differences in access to healthcare infrastructural resources, the growing burden of obesity and diabetes, rising healthcare costs and increased citizens’ knowledge had no predictive affect on the adoption of obesity and diabetes policy. We then turned to a subnational comparative analysis of the states of Mississippi in the United States and Rio Grande do Norte in Brazil to further assess the importance of infrastructural resources, at two units of analysis: the state governments versus rural municipal governments. Qualitative evidence suggests that differences in subnational healthcare infrastructural resources were insufficient for explaining policy reform processes, highlighting instead other potentially important factors, such as state-civil societal relationships and policy diffusion in Mississippi, federal policy intervention in Rio Grande do Norte, and politicians’ social construction of obesity and the resulting differences in policy roles assigned to the central government.

**Conclusion:** We conclude by underscoring the complexity of subnational policy responses to obesity and diabetes, the importance of combining resource and constructivist analysis for better understanding the context of policy reform, while underscoring the potential lessons that the United States can learn from Brazil.

## Introduction

The burgeoning growth of obesity and type-2 diabetes cases around the world has generated a considerable amount of scholarly attention. A problem has emerged in the literature, however: while several studies address the health causes and consequences of obesity and diabetes, little is known about the wider political, economic, infrastructural, and ideational constructivist context facilitating the pursuit of prevention and treatment policy at the national *and* subnational government level. The ongoing focus on national policy response has also overlooked the policy innovations that occur at the subnational government level and the contextual factors facilitating this process.

With these limitations in mind, the following research questions motivated this study. First, what does the literature say about the contexts facilitating national governments’ abilities to implement obesity and type-2 diabetes policies and is this literature supported with cross-national statistical evidence? Second, has this literature examined the contextual factors facilitating reform at the subnational government level: that is, state and rural municipal governments confronting an obesity and diabetes epidemic? And third, has this literature considered the extent to which the ideational construction of public health threats by policy-makers provides additional insight into the contextual factors facilitating policy reform?

To address these questions, this article introduces an analytical framework that combines a quantitative cross-national statistical and qualitative subnational comparative case study analysis assessing the importance of health, economic, and infrastructural resources as contextual factors facilitating policy-making, with an ideational constructivist analysis providing further insights into the political context facilitating this process. Building on McInnes,^1^ we argue that combining this empirical and constructivist approach provides a more thorough understanding of how context matters in the pursuit of obesity and type-2 diabetes policy at the national *and* subnational government level.

Most of the scholarly literature to date focuses on how the rise in obesity and type-2 diabetes prevalence rates and their associated ailments instigates fear and motivation for a national policy response.^1-3^ Others have instead argued that the high economic costs of obesity and diabetes create fiscal incentives for the pursuit of prevention programs.^1,4,5^ Alternatively, it is a government’s possession of hospital infrastructural and human resources that provide a favorable context for policy reform, while others instead underscore the public’s increased attention and pressures on government as conditions favorable for policy adoption.^6^

But to what extent are these theoretical approaches supported with cross-national statistical evidence? With the exception of the importance of infrastructural resources, such as the number of hospital beds and machinery, this study finds that most of this literature is not supported with such evidence.

In light of these statistical findings, we then turned to a comparative case study analysis of state governments and rural municipal governments in the United States and Brazil in order to further assess the importance of infrastructural resources as a contextual factor facilitating the adoption of policy, while exploring other potential factors facilitating policy reform at the subnational government level. In this study, state governments refer to state legislative assemblies and their health departments. By rural municipal governments, we refer to those locally elected governments that are distant from cities and typically underdeveloped. Specifically, we compared the state governments of Mississippi in the United States and Rio Grande do Norte in Brazil to rural municipal governments in these states: Holmes and Mossoró, respectively.

However, our comparison of the United States and Brazil at 2 levels of subnational government established little support for the importance of infrastructural resources as contextual factors facilitating policy reform. In Mississippi, findings suggest that while differences in access to infrastructural resources did account for differences in state government versus rural municipal government policy response, this was not the case in Rio Grande do Norte: notwithstanding having far fewer infrastructural resources, the municipality of Mossoró responded earlier than Rio Grande do Norte’s state government legislature and appeared to be more innovative in its policy response. We interpret this finding to suggest that there may be several alternative contextual conditions facilitating rural municipal governments’ abilities to be more progressive in pursuing obesity and type-2 diabetes policy.

Further research on Mississippi and Rio Grande do Norte suggested alternative conditions under which subnational governments pursue obesity and diabetes policy, factors that were nevertheless different for both nations. In Mississippi, state government policy diffusion and access to what we call *civic supporters*, ie, health officials’ usage of non-governmental organizations (NGOs) for information gathering and credibility facilitated the pursuit of policy reform. Conversely, in Holmes essentially no obesity and diabetes policies were pursued; this appears to be attributed to Holmes’ dearth of *civic supporters* and the absence of policy diffusion.

When compared to Brazil, however, none of the factors found in Mississippi’s state government appeared to be important in Rio Grande do Norte. Instead, Rio Grande do Norte’s reliance on the Ministry of Health’s (MoH’s) financial, technical, and human resource assistance appears to have been more important for facilitating the state legislature to respond to obesity, though not diabetes; Mossoró’s municipal policy response was also facilitated by this MoH assistance. Nevertheless, differences in the level of infrastructural resources and *civic supporters* between the state and rural municipal government level had no impact on policy response. While Rio Grande do Norte’s state government possessed more infrastructural resources and civic supporters, Mossoró had far fewer resources but was nevertheless able to respond earlier when compared to the state government. In fact, when compared to Holmes in the MoH, Mossoró appears to be more innovative in its policy response. As we discuss in the conclusion, this may provide an opportunity for Holmes and other rural municipal governments in the MoH to learn from Brazil.

Nevertheless, we combined our quantitative and qualitative analysis with an ideational constructivist approach underscoring additional contextual factors that further facilitated the introduction of policy. Building on McInnes,^1^ we argue that fully understanding the context of policy reform requires combining an analysis of material resource capacity with constructivist approaches to policy-making. According to this approach, policy actors frame healthcare issues in a manner that merges with broader, socially accepted ideas, and that because of this the idea gains influence and support for a particular policy path^1^; this occurs because the ideas used to frame a policy issue resonates with public understandings and therefore, has social legitimacy. Similarly in the United States and Brazil, we argue that political leaders framed obesity as an issue that comported with broader social and ideological conceptions of obesity that went beyond its medical scientific aspects. However, while this framing process helped to build a broad consensus for policy reform, political leaders in both nations eventually differed in the policy expectations and roles that they assigned to different levels of government, in turn leading to differences in favorable policy contexts and pathways.

In Brazil, presidents and senior health officials framed obesity as a public health threat reflecting ongoing poverty, poor nutrition, and the need to intervene on these grounds; at same time, obesity was framed as an issue affecting the nation’s international reputation, that is, one with a strong public health system that could avoid the challenges of obesity, ensure worker health and productivity. With politicians viewing the central government as responsible for taking the lead in building a stronger policy response, this kind of framing facilitated the MoH’s willingness to provide human resource and financial support to state and rural municipal governments. In the United States, the Barack Obama administration also framed obesity as a wider social problem, highlighting its association with poverty, under-nutrition, and inequality in access to physical fitness infrastructure. In contrast to Brazil, however, US politicians expected the state governments to play the dominant role in pursuing and implementing obesity policy; this engendered conditions that were not favorable for central government intervention at the subnational level.

## Methods

This study began during the spring 2013 and ended in the summer 2015. The principle researchers were located in the United Kingdom. All types of data analysis—cross national statistics and case studies—were conducted in the United Kingdom. Quantitative data was obtained from the World Health Organization (WHO) Global Health Observatory Data Repository, WHO World Health Statistics,^7^ the World Bank Data Repository, and the Central Intelligence Agency.

In this article, we adopted a nested approach to comparative research.^8^ This approach seeks to combine quantitative and qualitative analysis in order to confirm established theoretical frameworks. This methodological approach follows a particular sequence of events: theoretical frameworks are first examined in light of cross-national statistical evidence; next, if these frameworks are statistically confirmed, researchers employ case studies to further validate these statistical findings; if not, case studies are used to establish alternative hypotheses.^8^ If theoretical frameworks are statistically confirmed, and case studies validate statistical findings, the analysis concludes; if not, case studies are used to discover alternative hypotheses and outcomes.^8^ Thus, to effectively test for the importance of cross-national statistical findings, a nested approach requires the use of qualitative case studies to validate these findings with qualitative contextual analysis, or alternatively to use case studies to discover alternative approaches to policy reform.^8^ Indeed, in our study, comparing state and rural municipal governments in the United States and Brazil was done in order to further validate the efficacy of theories emphasizing the importance of infrastructural resources for the pursuit of obesity and type-2 diabetes policy, and to compare and explore alternative contexts facilitating the pursuit of these policies. Furthermore, examining the utility of an ideational constructivist approach to policy-making required the usage of qualitative case studies to illustrate this process.

The lead investigators collected data that best represented and that could be used to evaluate several theoretical frameworks discussing the types of context propitious for policy-making, such as the importance of type-2 diabetes and obesity prevalence and policies (denoted as *ObesePrev*/*Pol*; *DiabPrev*/*Pol,* respectively). We measured *ObesPrev* by the % of the population over the age of 20 with a body mass index (BMI) >20, while *DiabPrev* was measured by the total number of reported diabetic cases in each country. We assigned binary scores of 0 to 1 for *ObesePol* and *DiabPol,* which reflected the presence or absence of national policies; *gross domestic product (GDP)* was measured in US$, and government expenditures for health, *GovExp*, was measured as a % of total government expenditure for health, both proxies for government capacity to finance prevention and treatment services; the number of reported deaths in each country attributed to obesity and diabetes (*Deaths*) as factors potentially motivating governments to implement policy; the estimated total number of physicians and beds in each nation were selected (eg, *Physicians/Beds*) as proxies for health infrastructure and systems capacity, which signifies the ability to treat patients with obesity and diabetes-related illnesses; the variable *Pharma* represents the density of pharmaceutical personnel in each country, measured as a ratio of 1/1000, as an indicator of government capacity to provide medications; the ability of individuals to read (*Literacy*, measured by the total percentage of the country population able to read) and obtain information about obesity/diabetes prevention from the internet (*Internet*, measured by the total percentage of the population with access to the internet); the presence of national obesity/diabetes programs as a proxy indicator for government spending for non-communicable disease initiatives (*ExtNCD*, measured by binary scores of 0 to 1). Finally, we included binary scores for the presence or absence of a developed versus underdeveloped nations (*Developed*) and the presence of universal healthcare systems (*UHCs*) to account for other contextual factors facilitating the introduction of obesity and diabetes policy.

Cross-sectional data was collected for 83 country observations at different points in time (out of a total of 192 countries, though some values were missing for several years). With respect to qualitative data, information was obtained from published articles and government reports from Mississippi and Rio Grande do Norte. We also conducted in-depth 30-60 minute interviews with NGO leaders in Mississippi and health officials in Rio Grande do Norte during the summer of 2014, conducted via *Skype* telecommunications. Interviewees were randomly selected and gave consent to reveal their identities.

In this study, obesity and type-2 diabetes were selected because of their close epidemiological association.^9^ While hypertension, heart disease, and cancer are also related to obesity, we focused on obesity and diabetes because of the higher growth rate of these diseases relative to the former, and the projected expectation that type-2 diabetes will be the most prevalent global health threat in the future.^10^

With respect to the selection of country case studies, we purposefully selected state and rural municipal governments in the United States and Brazil with high levels of obesity and type-2 diabetes for the following reasons: first, to provide a more comprehensive analysis of the contextual factors leading to a policy response in different geographic regions at the subnational government level, rather than only focusing on one particular geographic area, eg, state government, which, in turn, would have provided a more limited form of subnational government analysis; second, to provide potentially new explanations for the contextual factors leading to the adoption of obesity and type-2 diabetes policy, which is a methodological advantage that scholars claim is associated with selecting case studies based on their known values on the dependent variable^11^; and third, to more effectively test our cross-national statistical findings with 2 geographic areas (ie, state and rural municipal governments) with high levels of obesity and diabetes, rather than just one geographic area in each subnational government.

Furthermore, the states of Mississippi and Rio Grande do Norte were chosen for several reasons. First, recent studies suggest that Mississippi has the highest rate of obesity and diabetes cases in the United States,^12^ while Rio Grande de Norte is among the top-ten most obese states in Brazil.^13^ Second, in both states high obesity and diabetes rates were prevalent at the urban and rural level. And while different in their political histories and institutions, cultures, and socio-economic conditions, in recent years Mississippi and Rio Grand do Norte were chosen because they were similar in the following respects: (1) both are democratic subnational governments with popularly elected governors and mayors; (2) both are poor states, with economic, per-capita income, and health indicators at levels lower than their respective national averages; (3) and finally, both states exhibit infrastructural and resource inequalities between the state and rural municipal government level. Within these states, the rural municipal governments of Holmes and Mossoró were selected because they were the rural governments in these states with the highest prevalence of obesity and type-2 diabetes cases with well-known infrastructural and human resource challenges. And finally, in general the United States and Brazil were selected because they are the 2 largest democracies in the Western Hemisphere with one of the highest obesity and diabetes prevalence rates.

Although Brazil has a UHC and a growing private insurance industry, the United States does not have a universal health insurance system but rather a patchwork of targeted insurance programs for particular segments of the population and a large private insurance sector. In contrast to the United States, moreover, in Brazil subnational governments rely more on the central government for federal grant assistant to fund specific public health programs and have a longer history of adopting federal technical policy norms and regulations governing healthcare.

## Results and Discussion

### Policy Responses to Obesity and Diabetes

Scholars have recently put forth several contextual factors facilitating the adoption of national obesity and type-2 diabetes policies. One school of thought emphasizes the burgeoning growth rate of obesity and diabetes cases and how this prompts government fear and policy reaction.^2,14^ The heightened spread of these diseases, especially among children,^3^ the physical and psychological complications associated with these ailments and the decreased quality of life has facilitated the implementation of national prevention programs.^3^

Others claim that it is the economic burden of obesity and diabetes that facilitates a national policy response. The escalating healthcare costs associated with treating these diseases,^9^ their related health conditions, such as cancer, high blood pressure, and heart disease motivates legislatures to pursue prevention and treatment policies.^4,15^ Direct costs for the government may not only include welfare benefits for the uninsured, but also payments for disability benefits.^5^ Yet another factor are the *indirect* costs associated with obesity and diabetes, such as the number of days lost from work due to illness, fatigue, lack of concentration at work, and learning impediments in school and low self-esteem.^5^

Still others emphasize the importance of governments’ access to infrastructural resources, human resources, such as hospitals, beds, medical equipment, supplies, and the availability of doctors and nurses in explaining the presence or absence of obesity and diabetes policies.^16-18^ Possessing these resources helps to reassure national legislatures that they have the capacity to implement policy. Acute differences in access to infrastructural and human resources has been seen as a reason for why state and urban governments are often more progressive in their policy response to obesity and diabetes when compared to rural governments.^16,17^

Finally, others claim that governments pursue the implementation of obesity and diabetes policies in response to a change in the national mood, fueled by citizens’ understanding and awareness of these health challenges.^6^ A sudden shift in citizens’ concern and heightened attention to a policy problem, often caused by a spike in media attention, can be sufficient for creating a “window of opportunity” and incentive for politicians to pursue legislation.^19^ This is because politicians are aware that citizens are aware of particular health threats; consequently, politicians have electoral incentives to respond to citizens’ concerns and pressures for policy reform.^6^

### Statistical Findings

With respect to the empirical efficacy of these theoretical approaches, results from a multivariable logistic analysis yielded mixed results. As Model 1 illustrates in the Statistical Appendix, with respect to the impact of obesity prevalence (*ObesePrev*), which as mentioned earlier affects politicians’ fears and incentives for reform, this does not appear to be significant in predicting the presence or absence of national obesity policy. The other variable that is indicative of the economic costs of obesity and diabetes, ie, *ExtNCD*; citizens’ knowledge, concerns and pressures for reform, ie, *Literacy* and *Internet* (access), also were not statistically significant (Table).

**Table:** Factors Contributing to the Adoption of National Obesity and Diabetes Policies

	**Model 1** **ObesePol**	**Model 2** **DiabPol**
ObesePrev	7.069 (4.386)	-5.723 (3.092)
ObesePol		4.272^c^ (1.660)
DiabPrev	5.112^a^ (1.516)	8.050 (3.025)
DiabPol	5.397^b^ (1.920)	
GDP	1.925 (1.176)	2.340 (2.383)
Physicians	-2.152^a^ (6.485)	-5.783 (1.608)
Beds	1.177^b^ (4.220)	-1.390 (2.650)
Pharma	1.301 (1.115)	-7.758 (1.013)
Deaths	-7.169 (3.938)	9.162^c^ (3.949)
ExtNCD	2.325 (1.333)	1.737 (1.773)
Literacy	-5.401 (4.316)	8.245 (5.460)
Internet	9.715 (1.308)	3.267 (6.286)
Developed	4.852 (2.261)	2.023 (1.790)
UHCs	3.490 (2.338)	1.570 (1.797)

Abbreviations: GDP, gross domestic product; UHC, universal healthcare systems. Reported coefficient estimates and standard errors (in parentheses). Null deviance: Model 1: 108.4 on 78 degrees of freedom; Model 2: 93.4 on 78 degrees of freedom; Residual deviance: Model 1: 47.1 on 65 degrees of freedom; Model 2: 45.5 on 65 degrees of freedom. Significance codes: ^a^ .01; ^b^ .01; ^c^ .05.

However, evidence does seem to suggest that a nation’s healthcare infrastructure is associated with the adoption of national obesity legislation. The indicator *Beds* had a positive coefficient estimate of 1.177 at the 0.001 level. The negative affect of *Physicians,* statistically significant at the 0 level but with a negative coefficient estimate of -2.152, is puzzling, however, suggesting that the presence of healthcare personnel has a negative influence on the adoption of national obesity policy. The independent variables *DiabPol*, ie, national diabetes policies, and *DiabPrev*, ie, the prevalence of diabetes cases, were also statistically significant at the 0.01 and 0 level, respectively; this may reflect governments waiting to implement obesity policies until after the emergence of an increase in diabetes cases and prevention policies, which may signify the gravity of the obesity situation, considering the strong association between obesity and diabetes.

Nevertheless, with respect to national diabetes legislation, none of the aforementioned theoretical frameworks proved to be statistically significant. As Model 2 illustrates, the only variable that was significant was *ObesePol*, ie, the presence/absence of national obesity policies, at the .01 significance level, with a positive coefficient estimate of 4.272. The presence of national obesity legislation may have generated incentives to introduce diabetes legislation, considering the likelihood that obesity increases the probability of acquiring diabetes. The variable *Deaths* was also statistically significant at the .01 level, suggesting that the number of fatalities associated with diabetes facilitated the introduction of national diabetes policies.

### Mississippi’s Response

In 2011, Mississippi was labeled as the most obese state in America,^12^ while the following year *The Economist* referred to Mississippi as “the fattest state in the fattest country in the Western World.”^20^ In 2007, approximately 30% of Mississippi residents were obese, while 1 in 10 individuals had diabetes.^21^ By 2011, an estimated 35% of Mississippi residents were obese, while an estimated 44% of children were overweight and/or obese.^22^ The number of diabetic cases also burgeoned. In 2010, Mississippi ranked second in the nation for having the highest level of type-2 diabetic cases, with 270 000 cases.^23^

Mississippi joins a myriad of poorer states in the southern Delta region exhibiting a high level of inequality between the urban and rural areas, not only in economic development, unemployment, and poverty, but also with respect to access to sound healthcare infrastructure, medicine, and primary care.^24^ Rural areas are also food desserts, such that access to affordable fruits and vegetables and grocery stores are minimal. Instead, much of these poorer areas are riddled with fast food restaurants, offering cheaper – though less healthier – foods. Underdevelopment in Mississippi’s rural landscape has also contributed to comparatively higher levels of obesity and diabetes when compared to more affluent urban centers.^25^ The poorer municipal governments of Holmes, Humphries, and Jefferson, for example, have had the highest level of obesity and diabetes in the state.^22^

In this context, Mississippi’s response to obesity and diabetes has varied at two levels of government: the state government, which is located in the urban area of Jackson county, versus the rural municipal government. At the state government level, since 2004 several program initiatives have been implemented by the governor and legislature; facilitating this process was the creation of the *Office of Healthy Schools* in 2004, which is located within the Mississippi Department of Education. With the support of the John D. Bower Foundation, this Office was created to help introduce and enforce bureaucratic regulations and legislative policies.^26^ In 2004, the state legislature implemented the “Local School Wellness Policy Guide for Development;” which helps schools to comply with federal guidelines and regulations. In 2006, the state legislature also ordered the Department of Education to clarify what could be sold in school vending machines, while mandating all schools to introduce wellness curriculum.^26^

State legislative efforts heightened with the introduction of the *Mississippi Healthy School Act* in 2007. This has been to date the most comprehensive legislative initiative addressing obesity in schools. Through this initiative, all schools boards are expected to have a minimum amount of time spent for physical education; to create school wellness programs; to employ a physical activity coordinator; to implement new regulations improving school breakfasts and lunches; to create school health advisory councils, while mandating that all schools have a nurse on staff to provide nutritional education and services.^26^

The state legislature has also implemented a myriad of diabetes prevention and treatment policies. In 2010, the Mississippi Department of Health created the *Mississippi Diabetes Prevention and Control Program* (MSDH). Through this initiative the state legislature works with the Department of Health and community-based organizations and schools to increase screening and primary care for diabetes, while funding initiatives that reduce sedentary lifestyles and various wellness programs.^23^ And in terms of access to medicine, since 1998 Mississippi’s Medicaid program has provided coverage for all diabetic medications.^27^ Mississippi joins only 2 other states, Missouri and Washington, where state insurance law requires diabetic coverage.^28^

Rural municipal government policy initiatives for obesity and diabetes are not as prevalent, however. For example, in the municipality with the highest level of obesity and type-2 diabetes, Holmes, health officials have not implemented any substantive policies for schools and communities. In addition to having high levels of unemployment, increased poverty and being a food dessert, Holmes is troubled by a dearth of healthcare infrastructure, eg, hospital beds and clinics, as well as physicians, nurses, and healthcare practitioners.^29^

In the absence of policy initiatives, civil society has played an important role. The *West Holmes Community Development Corporation* (CDC) has been helpful in addressing obesity and diabetes. The CDC provides support for increasing and sustaining agriculture production, youth employment and skills development in agriculture, and nutritional awareness to Holmes residents.^30^ With the CDC’s support, local vegetable markets have also opened.^30^ The *Delta Health Alliance* is another NGO that has worked with the Holmes, Caroll, Leflore, and Sunflower counties to organize awareness forums and to help mobilize community strategies tackling obesity, diabetes, hypertension, and other associated ailments.^31^ Other volunteer groups, such as the *Advocacy Academy*, has provided training on better nutrition and wellness to families and local churches.^32^ Finally, for several years Mississippi State University’s (MSU’s) Extension Service Program has been helping residents in Holmes, Carroll, Leflore, and Sunflower municipalities. Through MSU, workshops organized in these municipalities have increased awareness about obesity, diabetes, and heart disease, while planning community activities.^31^

In the absence of rural municipal government initiatives, the state government has been the only one capable of intervening and providing assistance through joint-partnerships with universities and community groups. In 2007, the Mississippi State Health Department established a partnership with MSU’s Extension Service Program, the Central MS Rural Development Network/Mallory Community Health Center, Holmes County School District, Southern Care Hospice and University of Mississippi Hospital Lexington campus to conduct the *Mississippi in Motion* initiative. By organizing focus group meetings in Holmes and other municipalities, this program aims to improve the health of those participating in the program through physical activity and improved nutrition.^33^ *Mississippi in Motion* has also organized local health fairs, where individuals’ BMI, blood pressure, and glucose and cholesterol tests are taken.

But what factors account for these different policy responses at the state versus rural municipal government level? Did the aforementioned theories emphasizing the importance of healthcare infrastructure matter in accounting for these outcomes?

In Mississippi, access to infrastructural resources appears to have facilitated the state legislature and bureaucracy’s response to obesity and diabetes, which was not, however, the case in Holmes.^25^ Moreover, the more developed urban areas of Jackson, Biloxi, and Gulfport have more hospitals, beds, and equipment when compared to Holmes and other poorer municipal areas.^25^

Nevertheless, the state government is also comparatively wealthier in terms of having access to *civic supporters*, ie, health officials’ access to NGOs that they can work with.^34^ Indeed, essentially all of the NGOs working with health officials are located in Jackson, where the state capital is located.^35,36^ The *Jackson Medical Mall Foundation’s Childhood Obesity Project*, the *Diabetes Association of Mississippi*, *Diabetes Support Group*, and *Mississippi Health First* are a sample of NGOs working with state health officials on obesity and diabetes policies.^37,38^ The state health department in Jackson has worked closely with these organizations in order to obtain information about the obese and diabetic community’s needs, while organizing conferences, conducting health surveys, and designing policy interventions.

Conversely, there is a dearth of NGOs in Holmes.^39,40^ While universities have been present, and while there are some select NGOs that provide rural medical care, such as the *Delta Health Alliance* and *Advocacy Academy*, when compared to Jackson, Holmes has far fewer NGOs that local officials can work with^41,42^; this has made it difficult for these officials to incorporate the healthcare needs of Holmes residents.^43^

Furthermore, understanding how the obesity epidemic was politically and socially constructed and the policy expectations that ensued helps to provide additional insight into the contextual challenges that Holmes encountered. Entering office in 2008, First Lady Michelle Obama emphasized the importance of responding to the obesity epidemic, especially among children. In making her claim, she underscored how rising levels of poverty, poor nutrition, and inequality in access to parks and physical fitness opportunities contributed to the epidemic.^44^ Her aim was to reveal obesity’s broader socio-economic significance and to use this as a justification for more policy action.^44^ Through these efforts, Michelle Obama was able to win widespread support for the passage of her 2010 *Let’s Move* legislation. However, through this endeavor, which entailed federal regulations requiring the introduction of healthy foods in schools, greater awareness and community involvement, she called on the state governments to take the lead in creating and implementing policy.^44^ In so doing, state and rural municipal governments were left on their own, with little federal support amidst fiscal budget cuts in several states.^45^ Thus, Michelle Obama’s political and social construction of obesity was not followed up with policy roles and expectations facilitating—and indeed encouraging—the federal government’s assistance to local governments. This context imposed a major burden on poorer rural municipal governments, such as Holmes.

But what other factors accounted for the state government’s more successful response? Policy diffusion also seems to have been important. During Mississippi Governor Hayley Barbour’s administration (2004-2012), there was interest in learning how other states responded to obesity and diabetes. In an effort to strengthen his state’s response, “they [governor Barbour’s office] examined initiatives implemented by other states, reviewing much of the legislation that had been introduced in 45 states during 2005 to address the increasing rate of childhood obesity, as well as similar action taken through regulation and policy.”^46^ Mississippi’s state government therefore seems to have pursued legislation that was popular and seemingly effective in other states. The social context within which policy diffusion emerged was also favorable. By 2005, surveys conducted by the *Centre for Mississippi Health Policy* revealed that most Mississippians realized their dire situation and that they needed a larger public sector role in combating obesity.^26^

### Rio Grande do Norte’s Response

Similar to Mississippi, Rio Grande do Norte in Brazil is a leading state in terms of the prevalence of obesity and diabetic cases. In fact, estimates suggest that the state is in the top-10 for obesity prevalence.^13^ Similar to Mississippi, obesity prevalence is high in urban and rural areas. In the state capital, Natal, in 2006, the percentage of obese individuals with a BMI >30 kg was 13.1% of the population, while the percentage of overweight (BMI >25 kg) was 43.3% of the population^47^; moreover, by 2012, the percentage of obese individuals had increased to 21.2% while the percentage of overweight increased to 52.2%.^47^ At the same time the rural municipality of Mossoró, which is located about 275 kilometers northwest from Natal, had an estimated 40% of obese individuals in 2012.^48^ Diabetes is also highly prevalent. In Mossoró, in 2011 there were an estimated 11 000 diabetics.^49^

When compared to Mississippi, Rio Grande do Norte’s state government response was considerably more delayed and half hearted at best. Despite the government’s knowledge of growing obesity and diabetic cases, no policy efforts were made until 2012. That year, state legislative representative Larissa Rosado (PSB political party) succeeded in approving the *Semana Estadual de Combate á Obesidade Infantil* (State Week to Combat Infantile Obesity). Through this initiative, eventually promulgated through Law No. 82 in 2013, the state government supported prevention campaigns seeking to increase awareness of obesity, especially among children, while supporting the provision of nutritional and physical education classes in schools.^50^ Yet, the state has not pursued any other legislation for obesity and diabetes.^51^

But what were the factors facilitating the state government’s policy response? It seems that this response was facilitated not by the growing prevalence of obesity and diabetic cases and their projected healthcare costs, but by the governor and state legislature’s support from the federal government.^51^ Despite possessing sound healthcare infrastructure, physicians, and other medical staff,^52^ the state government seems to have expected and depended on receiving support from the MoH for its obesity policies.^51^ In 2011, for instance, the MoH provided R$ 6.2 million reais to Rio Grande do Norte’s state treasury to support the funding of obesity prevention programs.^53^

Furthermore, the MoH provides human resource support to Rio Grande do Norte’s health department. Through the Family Health Program (FHP), the MoH provides physicians, nurses, and nutritionists that travel to health centers and schools throughout Rio Grande do Norte.^54^ The FHP teams help by providing courses on better nutrition, wellness, and diabetic treatment.^54^

In a context of limited funding for healthcare, this additional financial and human resource assistance from the center seems to have been a key factor facilitating the governor and state legislature’s pursuit of the aforementioned 2012 legislation.^51^ This federal support provided state legislators with the confidence and reassurance needed to implement policy, as this support supplied the funding and the additional manpower needed to provide healthcare services for obesity and diabetes.^51^

But what about the contextual factors facilitating Mississippi’s governor and state legislature ability to pursue policy, such as the governor’s interest in policy diffusion? No evidence suggests that policy diffusion was important for Rio Grande do Norte’s Governor, Rosalba Ciarlini, and the state legislature. Alternatively, did access to infrastructural resources play an important role, as seen in Mississippi? Rio Grande do Norte’s state government did have access to ample infrastructural resources prior to the aforementioned 2012 legislation.^52^ Though to a lesser extent, these infrastructural resources were also present in other cities.^52^ Yet, in contrast to what we saw in Mississippi, research suggests that these resources were not important in facilitating the state of Rio Grande do Norte’s government pursuit of policy reform.^51^

It may nevertheless be the case that access to *civic supporters* helps to explain Rio Grande do Norte’s response. In contrast to what we seen in Mississippi, however, there have not been any obesity and diabetes NGOs working with Rio Grande do Norte state health officials. There is only one NGO focused on obesity, the *SPK Movement*, which was created by Patricia Motta in 2011.^55^ *SPK* provides a gym for residents in the city of Natal and offers classes on nutrition and physical fitness.^55^ In contrast to what was seen in Mississippi, *SPK* appears to work on its own rather than with state health officials or even universities to provide these services. And with respect to diabetes, while the *Fórum de Atualização e Educação em Diabetes* (Forum for the Actualization and Education of Diabetes) was created in 2011, it works independently from the state government, organizing events with volunteer members and private medical clinics.^56^ There are no NGOs working on diabetes.

Nevertheless, how did the rural municipal government of Mossoró in Rio Grande do Norte respond to obesity and diabetes? Was Mossoró just as delayed and lackluster in its policy response?

Interestingly, although also delayed its policy response, evidence suggests that Mossoró was somewhat more innovative. For example, by 2011, the municipal legislature created the *Centro de Apoio ao Controle da Obesidade Jensen Jefferson Diógenese e Medeiros* (Jensen Jefferson Center for Support and Control of Obesity).^48,57^ This center focuses on the prevention of obesity, providing classes from better nutrition to having healthier lifestyles, and psychological counseling, particularly depression.^48^ The Center has a large staff, with 2 nutritionists, 2 physical education instructors, and 1 social assistant providing daily counseling services.^58^

Seeking to further increase public awareness and provide assistance, in 2011 Mossoró legislator Francisco José Jr. also passed legislation providing funding for obesity public media campaigns; moreover, he went as far as to create Mossoró’s very own “week of focus on obesity.”^59^ And in 2012 the municipal legislature created the *Programa Municipal de Prevenção da Obesidade em Criancas e Adolescentes* (Municipal Program for the Prevention of Obesity in Children and Adolescents).^60^ This policy provides funding for schools to create nutritional programs for families and children, gradually helping change daily eating habits while providing suggestions for physical exercise routines.^60^

When it came to diabetes, however, Mossoró joined its state government in not having an effective policy response. To our knowledge, there exist no legislative efforts to provide diabetic prevention and treatment services. While health officials do warn about the likelihood of obtaining diabetes due to weight gain through the aforementioned public media campaigns, there are no specific policy initiatives for diabetes. Furthermore, all medications for diabetes are financed and provided by the national MoH.^61^

As we saw at the state government level, Mossoró has also benefited from federal financial and human resource assistance. Through the aforementioned FHP, FHP teams provide obesity and diabetes services to families and schools. In a context where there is a limited amount of infrastructural and human resources,^52^ this support has been important. Additionally, through the MoH’s *Programa Saúde na Escola* (PSE) program, Mossoró’s health department receives a quarterly stipend of R$ 395 000 reais^53^; this money is conditional, based on the municipal health department’s success in effectively using the money and adherence to PSE guidelines.^53^

Proactive federal support amidst limited infrastructural resources seems to have provided the contextual context needed to facilitate Mossoró health officials’ pursuit of obesity legislation in 2011. It is also important to note that this partnership with the federal government dates back to 2007.^62^ In light of this history, there was an expectation among Mossoro’s health officials that they could continue to expect this federal assistance.

The federal government’s framing of the obesity epidemic also facilitated this support. With the arrival of President Luiz Inácio Lula da Silva in 2003, the president and senior health officials began to emphasize the broader socioeconomic significance of obesity, politically and socially constructing the epidemic as an issue of ongoing poverty, under-nutrition, and inequality in access to healthier foods, recreational parks, and physical fitness.^63^ By constructing obesity in this manner and working with like-minded participatory institutions within government bringing together health officials, activists, and the private sector working on obesity policy, Lula was able to win their support when pursuing a more aggressive central government response.^64^

At the same time, senior health officials viewed obesity as an international reputational issue. For example, the Minister of Health under Lula, José Gomes Temporão, and other senior officials emphasized that Brazil’s international reputation as a healthy and prosperous nation, especially on the eve of the World Cup and Olympics, was vital for securing the nation’s image as a prosperous emerging economy; in this regard, these leaders loathed the idea of being seen as similar to the United States in confronting and obesity epidemic.^65^ Instead, the central government wanted to ensure that it could build a strong public health response to obesity, and that it could work closely with subnational governments to achieve this goal.^65^

But what about the issue of infrastructural resources and policy diffusion? Evidence suggests that none of these factors were important. At no point did the mayor of Mossoró, Maria de Fátima, or her legislative representatives look to other municipal or state governments for policy inspiration. Infrastructural resources also could not have been a factor. Mossoró lacked a sufficient amount of hospitals, beds, and medical staff, especially when compared to larger cities.^52^

And what of the Mossoró health department’s access to *civic supporters*? While there have been NGOs focused on diabetes, none exist for obesity. In Mossoró, there is a civic movement called the *Fórum de Atualização e Educação em Diabetes* (Forum for the Actualization and Education of Diabetes). The *Fórum’s* focus is to increase awareness, screening, and to provide counseling and treatment for newly discovered diabetic patients.^56^ Furthermore, every November the *Fórum* organizes a public event to reach out to the community.^56^ While new and small in membership,^56^ the *Fórum* is comprised of activists and medical doctors. Yet, the *Fórum* has been more likely to partner with other NGOs, such as the *Rotary Club of Mossoró*, rather than with municipal health officials. Consequently, there are few opportunities for municipal officials to use the *Fórum* for their policy endeavors. Nevertheless, as the *Fórum* grows, this may provide more opportunity and incentive for greater collaboration between them.

## Conclusion

Conducting a nested analytical approach to exploring the contextual factors facilitating national and subnational governments’ pursuit of obesity and diabetes policy provided intriguing empirical results. At the cross-national statistical level, evidence suggests that of the aforementioned theoretical schools of thought examined, only infrastructural resources correlated with the adoption of national obesity policy. This finding provided fertile ground to then assess the importance of infrastructural resources at the subnational government level.

Evidence from Mississippi did support the importance of infrastructural resources, accounting for the context facilitating the state government’s earlier policy response when compared to Holmes. Nevertheless, this case also revealed the limitations to an infrastructural approach, suggesting that other factors were equally as important, such as the usage of *civic supporters*. Indeed, Mississippi’s department of health worked closely with several NGOs, providing them with important information while legitimizing officials’ policy efforts, though this was not the case in Holmes. Moreover, policy diffusion appeared to further facilitate the pursuit of obesity policy. In contrast to Mississippi, in Rio Grande do Norte infrastructural resources did not facilitate the adoption of policy at the state and rural municipal government level. And even after possessing fewer infrastructural resources when compared to Rio Grande do Norte’s state government, Mossoró still introduced innovative obesity and diabetes policies.

But were Mississippi’s alternative hypotheses, such as *civic supporters* and policy diffusion, also important in Rio Grande do Norte? The presence of NGOs does seem to have facilitated Rio Grande do Norte’s state legislature’s adoption of obesity legislation in 2012. Conversely, while NGOs were present in Mossoró, they were distant from municipal health officials, thus failing to become a resourceful partner. Consequently, the importance of civic supporters does not appear to have been as important in Rio Grande do Norte, as Mossoró’s officials were able to respond earlier and in a more innovative manner without them. How about policy diffusion? In further contrast to Mississippi, this was also not an important factor in Rio Grande do Norte.

Instead, our comparison of the United States and Brazil revealed the importance of alternative and more favorable contextual factors unique to Brazil: that is, state and rural municipal government reliance on federal financial and human resource assistance. At the state and especially rural municipal government level, receiving federal grant assistance and additional healthcare workers, mainly through the FHP program, appeared to provide legislators and health officials with the confidence and incentives needed to implement policy.

Building on McInnes,^1^ we nevertheless found that providing an analytical framework that combines our analysis of material resource capacity with an analysis of the ideational construction of obesity and diabetes helped to further advance our understanding of the contextual factors facilitating policy-making while highlighting additional differences between the United States and Brazil. While political leaders in both nations viewed these health threats as politically and socially important, Brazil’s leaders were the only ones to assign expectations on the federal government to provide assistance to state and especially poorer rural municipal governments in order to ensure that policies were effectively implemented. Using this constructivist approach therefore revealed that our contextual analysis of the importance of infrastructural and other types of resources is limited in its ability to fully account for the contextual factors facilitating policy reform at the subnational level.

The case of Mossoró in Brazil also suggests that poorer municipal governments can be important incubators of policy innovation, and that their isolated context may instigate local government incentives to implement innovative policy. Nevertheless, Mossoró’s success also suggests that rural municipal governments in the United States seeking to find ways to better respond to obesity and diabetes may stand to gain from partnering with municipal policy-makers in Brazil and learning from their experiences. The fact that Mississippi’s health officials have recently reached out to Iranian health officials in order to learn about innovations in rural primary healthcare^66^ suggests that learning from Brazil’s rural municipal governments can be possible. Researchers will need to explore this possibility and to what extent Brazil and other emerging nations’ innovative policy ideas can be adopted in the United States.

## Acknowledgements

The author wishes to thank James McGuire, Daniel Kaufman, Patricia Cioricci, and Adam Okulicz-Kozaryn for excellent comments during the drafting of this article.

## Ethical issues

No ethical review was required because this study did not obtain primary data from human subjects with respect to their particular health status.

## Competing interests

Author declares that he has no competing interests.

## Author’s contribution

EJG is the single author of the paper.

## Key messages

**Implications for policy makers**

- Research in this article suggests that policy-makers need to examine the context of obesity and diabetes policy at the national and subnational government level.
- Policy-makers need to combine their public discussion of obesity and diabetes with greater central government roles assisting state and especially poor rural municipal governments.
- Policy-makers need to work closely with rural municipal governments to provide favorable financial and infrastructural contexts facilitating policy-making.
- Policy-makers at the state and rural municipal government level need to work closely with non-governmental organizations (NGOs) to obtain the information needed to devise effective prevention and treatment policies.

**Implications for public**

The adoption of policy lessons from this article could benefit the public by providing additional contextual information to policy-makers on how to improve their prevention and treatment services for obesity and diabetes programs. Diabetics and those struggling with obesity could, for example, benefit from politicians’ matching their public discussion of these ailments with enhanced roles for central and state governments as well as the provision of additional central government financial assistance to rural municipal governments.
